# Electron Microscopy in Renal Biopsy Interpretation: When and Why It Still Matters

**DOI:** 10.7759/cureus.96311

**Published:** 2025-11-07

**Authors:** Hussein Qasim, Zeina Hayajneh, Karis Khattab, Matteo Luigi Giuseppe Leoni, Giustino Varrassi

**Affiliations:** 1 Department of Pathology and Laboratory Medicine, Jordan University of Science and Technology, Irbid, JOR; 2 Department of Public Health, Jordan University of Science and Technology, Irbid, JOR; 3 Faculty of Medicine, Jordan University of Science and Technology, Irbid, JOR; 4 Department of Medical and Surgical Sciences and Translational Medicine, Sapienza University of Rome, Rome, ITA; 5 Department of Pain Medicine, Fondazione Paolo Procacci, Rome, ITA

**Keywords:** c3 glomerulopathy, electron microscopy, focal segmental glomerulosclerosis, glomerular diseases, membranous nephropathy, renal biopsy, ultrastructural pathology

## Abstract

Electron microscopy (EM) remains an indispensable tool in renal pathology, providing ultrastructural details that complement light microscopy (LM) and immunofluorescence (IF) in the diagnosis of glomerular diseases. This review outlines the diagnostic role of EM across major categories of glomerular pathology, emphasizing its value in confirming and refining histopathological diagnoses. In membranous nephropathy, EM visualizes subepithelial electron-dense deposits and tracks disease progression through glomerular basement membrane (GBM) remodeling. In immune complex-mediated glomerulonephritides, such as IgA nephropathy and lupus nephritis, EM localizes deposits to mesangial, subendothelial, or subepithelial regions, facilitating accurate classification. For C3 glomerulopathies, EM distinguishes dense deposit disease from C3 glomerulonephritis by revealing unique ribbon-like intramembranous densities. In hereditary nephritides like Alport syndrome and thin basement membrane nephropathy, EM provides pathognomonic findings of GBM lamellation and thinning that often precede or guide genetic testing. Additionally, EM identifies organized deposits in fibrillary and immunotactoid glomerulopathies and helps differentiate diabetic glomerulosclerosis from mimickers through assessment of GBM thickening and matrix expansion. Despite advances in molecular diagnostics, EM remains essential for identifying subtle ultrastructural changes, validating immunopathologic interpretations, and guiding clinical management. This article reinforces EM’s continued relevance in an era of expanding genetic and serologic tools.

## Introduction and background

Electron microscopy (EM) has been integral to renal pathology for over six decades [1]. In the 1950s, the advent of immunofluorescence (IF) and transmission EM revolutionized kidney biopsy analysis, revealing ultrastructural details invisible to light microscopy (LM) [2]. Early ultrastructural studies defined classic lesions, for example, EM demonstrated the diffuse podocyte foot process effacement (fusion of podocyte cell extensions seen in nephrotic syndromes) underlying minimal change disease (MCD), a lesion that appears nearly normal by LM [3]. Since then, EM has been routinely used alongside LM and IF in a correlative triad to evaluate native renal biopsies [3]. By providing nanoscale resolution of glomerular basement membranes (GBMs) (the filtration barrier between blood and urine), cells, and deposits, EM yields critical diagnostic information in many glomerular diseases [4]. Historically, multiple studies have quantified EM’s diagnostic contribution [5]. Despite its demonstrated value, the use of diagnostic EM has faced challenges [6]. EM is costly and labor-intensive, requiring expensive equipment and specialized technologists [1]. Some centers under budget pressures have debated restricting EM to select cases, especially as immunohistochemistry (IHC) (antibody-based tissue staining technique) and molecular tests emerge for diseases once diagnosed only by EM [7]. Nonetheless, expert renal pathologists emphasize that failure to perform EM in certain cases will result in missed or incorrect diagnoses with potential clinical consequences [1]. This review provides a comprehensive update on the enduring importance of EM in renal biopsy interpretation. We outline how LM, IF, and EM complement each other, review disease-specific ultrastructural features, discuss the diagnostic yield of EM, and address current challenges and future developments. Practical recommendations are included to guide clinicians and pathologists on when and how to optimally utilize EM in the evaluation of renal biopsies. While EM remains an invaluable adjunct in renal pathology, it is not universally required for every kidney biopsy. In many clinical settings, particularly resource-limited centers, accurate diagnosis can often be achieved using high-quality LM with special stains, IF, and appropriate clinical correlation.

## Review

Methods

This article is a narrative literature review rather than a systematic or meta-analytic study. A structured search was conducted using PubMed, Scopus, and Google Scholar for English-language publications between 1990 and 2025. Search terms included combinations of “electron microscopy,” “renal biopsy,” “glomerulonephritis,” “glomerular disease,” “ultrastructure,” and “kidney pathology.” Both classic foundational studies (defining ultrastructural lesions in the 1950s-1990s) and recent publications addressing modern applications, diagnostic yield, artificial intelligence (AI), and molecular correlations were included.

Studies were included if they described diagnostic, prognostic, or technical aspects of EM in renal pathology or compared EM with LM/IF findings. Exclusion criteria were non-human studies, purely technical physics papers on microscopy, and articles without clinical or pathological relevance.

Because this review aimed to provide a descriptive synthesis of the diagnostic value of EM rather than a quantitative comparison, risk-of-bias assessments and meta-analytic pooling were not applicable. Evidence was integrated thematically across disease categories (podocytopathies, immune complex diseases, complement-mediated diseases, hereditary nephropathies, and transplant pathology).

Complementary roles of LM, IF, and EM in kidney biopsy interpretation

A renal biopsy is best understood as a composite puzzle: LM, IF, and EM each provide unique pieces that together yield a complete diagnostic picture [8]. LM (typically with H&E, periodic acid-Schiff (PAS), trichrome, and silver stains) reveals glomerular architecture, patterns of injury (proliferation, sclerosis, crescents), and interstitial and vascular changes [9]. However, LM alone often lacks specificity; different diseases can appear similar by LM [10]. IF microscopy (or IHC in some labs) detects immunoglobulin and complement deposits, enabling classification of glomerulonephritides by immune complex content (e.g., “full-house” Ig in lupus, dominant IgA in IgA nephropathy) [11]. Yet IF localizes deposits only in a general way (mesangial vs capillary wall) and may be negative in some entities (e.g., C3 glomerulopathy (C3G) or forms of pauci-immune disease) [12]. EM adds a critical third dimension by visualizing the ultrastructural location and nature of deposits, as well as subtle changes in cellular organelles and GBM structure [13]. EM can confirm findings suggested by LM/IF or uncover occult lesions that are missed by other modalities [14].

In most biopsy specimens, routine stains (H&E, PAS, trichrome, and silver) and direct immunofluorescence (DIF) for immunoglobulin and complement components provide sufficient information for accurate diagnosis and disease classification [12]. EM should therefore be viewed as a supplementary and confirmatory tool, particularly valuable when LM and DIF findings are equivocal or conflicting [14]. This balanced triad (LM, DIF, and EM) ensures maximal diagnostic precision while maintaining cost-effectiveness in routine practice [14].

**Figure 1:**
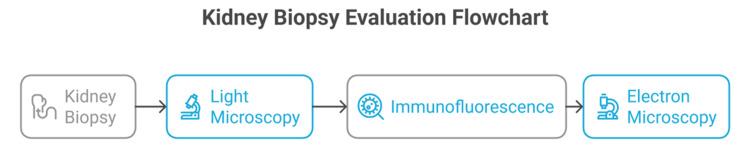
Kidney biopsy evaluation flowchart The figure is original and created by the authors. Credit to: Karis Khattab. All tools used in this study were free to use.

The diagnostic workflow of kidney biopsy evaluation is illustrated in Figure 1, highlighting the sequential and complementary use of LM, IF, and EM.

For example, in nephrotic syndrome, LM may be unremarkable in a child’s biopsy; IF may be negative. In such a case, EM is indispensable to confirm diffuse podocyte foot process effacement and establish MCD [15]. Conversely, an adult with nephrotic syndrome might show thickened capillary loops by LM and granular IgG-C3 along the GBM by IF; EM will demonstrate subepithelial immune deposits confirming membranous nephropathy (MN) [16]. In immune-complex glomerulonephritis (GN) (like post-infectious GN), IF classically shows “starry sky” granular deposits; EM then reveals large subepithelial “hump” deposits corresponding to that pattern [17].

Minimal change disease and early FSGS: the podocytopathies

**Figure 2:**
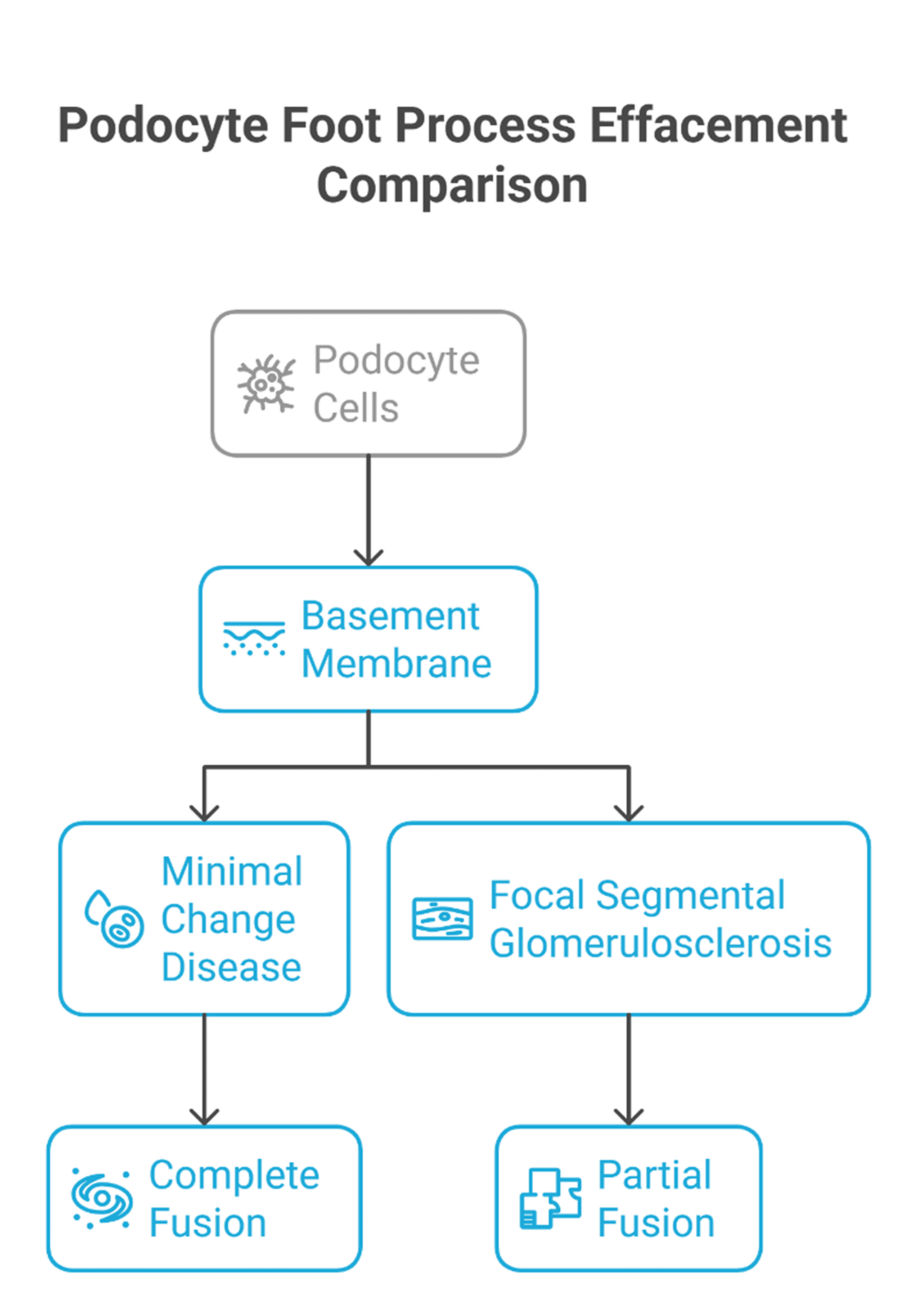
Podocyte foot process fusion patterns difference between minimal change disease and focal segmental glomerulosclerosis The figure is original and created by the authors. Credit to: Karis Khattab. All tools used in this study were free to use.

MCD is defined by nephrotic-range proteinuria with glomeruli that appear nearly normal by LM (“minimal change”) [18]. IF is typically negative or shows only minimal nonspecific IgM or C3 trapping [19]. On EM, the interdigitating foot processes that normally appear as discrete “peg-like” projections are replaced by a continuous layer of fused podocyte cytoplasm covering the GBM (“foot process fusion”) [20]. The GBM itself remains of normal thickness and texture in pure MCD [20]. In some cases, EM may also show vacuolization or microvillous transformation of podocyte cell surfaces, reflecting podocyte injury [21]. Diffuse foot process effacement on EM is required to confirm MCD, especially in children, where MCD is the most common cause of nephrotic syndrome, and routine EM is advised to avoid missing early FSGS [22]. Podocyte foot process fusion patterns differ between MCD and focal segmental glomerulosclerosis (FSGS), as summarized schematically in Figure 2.

FSGS, especially in its early or primary (idiopathic) form, can overlap with MCD [23]. Patients often present similarly with nephrotic syndrome [23]. By LM, FSGS is defined by segmental sclerosis in some glomeruli, but in early cases, the sampled glomeruli might all appear normal (leading to a diagnosis of “MCD” unless deeper levels or repeat biopsy find a sclerotic focus) [24]. EM in primary FSGS typically shows extensive foot process effacement indistinguishable from MCD [3]. In both MCD and primary FSGS, >80% of the capillary loop surface may be denuded of slit diaphragms [21]. Subtle clues favoring incipient FSGS can include more pronounced podocyte microvillous transformation, cytoskeletal reorganization (microfilament condensation), or rare segmental areas of GBM wrinkling on EM [25]. However, EM alone cannot reliably distinguish MCD from early FSGS if no sclerosis is seen by LM [26]. Therefore, thorough LM examination of multiple levels is essential [27]. In practice, the term “MCD” is used if no sclerosis is found, and EM shows diffuse foot process fusion; “FSGS” is diagnosed if even one segmental lesion is present by LM [28]. Both are considered podocytopathies, and EM confirming foot process effacement is critical to exclude other causes of proteinuria [29]. Notably, secondary FSGS (due to hyperfiltration, viral injury, etc.) may show only partial foot process effacement on EM (e.g., 50-80%), whereas primary FSGS usually mirrors MCD with near-complete effacement [30]. Thus, EM can also help differentiate primary (diffuse effacement) from secondary (patchy effacement) FSGS in context [31]. A comparative summary of ultrastructural differences is provided in Table 1.

**Table 1:** Electron microscopy (EM) features differentiating minimal change disease (MCD) and focal segmental glomerulosclerosis (FSGS)

Feature	Minimal Change Disease	Focal Segmental Glomerulosclerosis
Foot process effacement	Diffuse (>90%) [32]	Segmental [33]
Immune deposits	None [34]	May have segmental IgM/C3 [35]
Glomerular basement membrane changes	Normal [32]	Segmental wrinkling or collapse [33]
Tubuloreticular inclusions	Rare [34]	Possible in secondary forms [35]

Membranous nephropathy

MN is a common cause of adult nephrotic syndrome characterized by subepithelial immune complex deposition along the GBM [36]. Classic LM findings are thickened capillary walls with “spikes” on silver stain, and IF shows finely granular IgG and C3 along GBM [37]. EM clinches the diagnosis by directly visualizing the subepithelial electron-dense deposits and their morphological effects on the GBM [38]. On EM, the deposits appear as round or oval osmiophilic granules on the outer (subepithelial) surface of the GBM, often measuring 100-1000 nm [39]. In early-stage MN (Stage I), these deposits sit on an otherwise normal GBM [40]. In Stage II, the GBM between deposits begins to react by laying down new matrix, forming the “spikes” that protrude between immune deposits - EM shows these spikes as intervening projections of GBM between the electron-dense deposits [41]. By Stage III, deposits become incorporated within a thickened GBM, and in Stage IV, many deposits have been resorbed, leaving an irregularly thickened, sclerotic GBM [42]. Throughout these stages, EM typically shows diffuse foot process effacement overlying the deposits (explaining the heavy proteinuria) [43].

EM not only confirms the presence and location of immune deposits but can also help in challenging cases [44]. For instance, early or “incipient” membranous may show only subtle GBM changes by LM, and IF can sometimes be equivocal (especially if tissue was formalin-fixed and IgG eluted) [45]. EM can detect small scattered subepithelial deposits even when IF staining is weak, establishing the diagnosis of MN [46]. In lupus patients, EM can distinguish idiopathic MN from lupus class V by identifying additional mesangial or subendothelial deposits if present (favoring lupus) [47]. An important advance in recent years has been the identification of phospholipase A2 receptor (PLA2R) as the antigen in primary MN [48]. While serologic or immunohistochemical tests for PLA2R can now support the diagnosis of primary MN, EM still plays a role, for example, confirming that deposits are indeed subepithelial and not intramembranous or mesangial, and assessing chronic damage (GBM remodeling), which has prognostic value [49].

**Figure 3:**
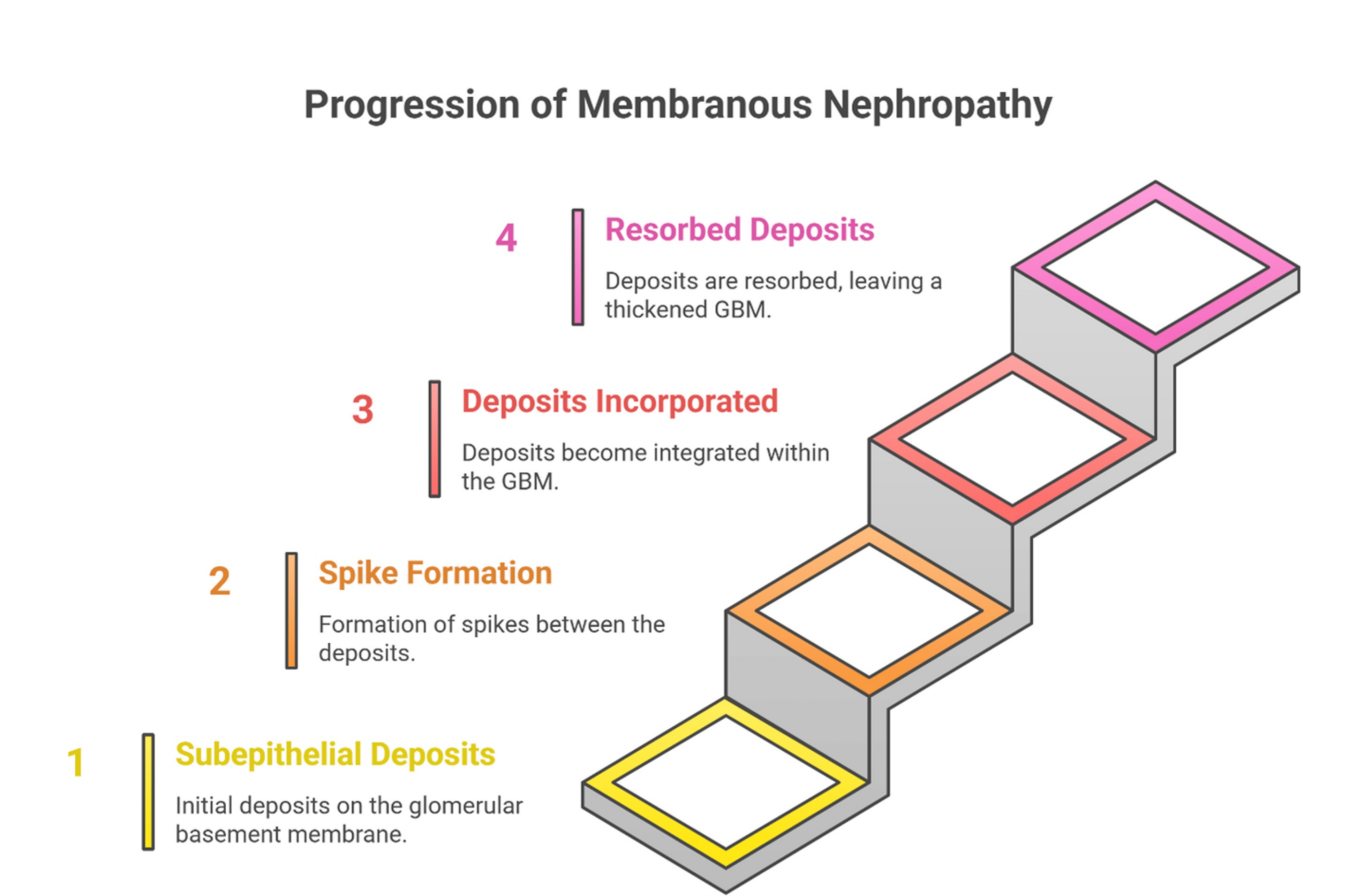
Progressive morphological changes of the glomerular basement membrane in membranous nephropathy The figure is original and created by the authors. Credit to: Karis Khattab. All tools used in this study were free to use.

Overall, EM in MN not only confirms the diagnosis but also assists in staging and assessing chronicity. The next section discusses immune complex-mediated glomerulonephritides, where EM’s localization of deposits refines disease classification (Figure 3).

IgA nephropathy, lupus nephritis, and other immune complex glomerulonephritides

Immune complex-mediated glomerulonephritides encompass a range of diseases (IgA nephropathy, lupus nephritis (LN), post-infectious GN, membranoproliferative GN, etc.) in which the deposition of immunoglobulins and complement in glomeruli incites injury [50]. IgA nephropathy (IgAN) is the most common primary GN worldwide [51]. By LM, it often shows mesangial proliferative GN, and IF is diagnostic with dominant IgA deposits in the mesangium [52]. EM in IgAN typically reveals electron-dense deposits primarily in mesangial areas [53]. These deposits are often patchy and have moderate electron density. In more severe IgAN, EM can also show subendothelial deposits, especially in IgA-driven crescentic GN or combined IgA/membranoproliferative GN (MPGN) patterns [54]. Small subepithelial deposits (“humps”) can occur in IgAN after infections but are less common [54].

LN, as part of systemic lupus erythematosus, produces a spectrum of immune complex deposition patterns (Classes I-VI) [55]. IF is usually “full-house” (IgG, IgA, IgM, C3, C1q all positive), and EM is invaluable for mapping the location and abundance of deposits, which guide classification [56]. In Class III/IV (proliferative LN), EM shows abundant deposits in subendothelial locations (large “wire-loop” deposits) as well as mesangial deposits [57]. These subendothelial immune complexes correlate with the severe endothelial injury and often with “hyaline thrombi” by LM [57]. In Class V (membranous LN), EM demonstrates a pattern identical to idiopathic membranous-subepithelial deposits with GBM spikes - usually accompanied by mesangial deposits (which help distinguish from primary MN) [58]. Lupus often also features tubuloreticular inclusions (TRIs) within glomerular endothelial cell cytoplasm, visible by EM as lattices or fingerprint-like tubular structures ~25 nm in diameter [59]. TRIs are induced by high interferon-alpha levels, and while not specific to lupus (they can appear in HIV or other interferon states), their presence in the right context supports lupus activity [60]. In fact, the “full house” IF and TRIs on EM strongly favor LN over other immune complex GN [61]. EM can also identify organized deposits in lupus (e.g., cryoglobulin with microtubules or fingerprint structures) that indicate mixed pathologies [62].

Post-infectious (exogenous immune-complex) GN, classically post-streptococcal GN, shows by EM the large distinctive subepithelial “hump” deposits - extremely electron-dense dome-shaped deposits on the outer GBM [63]. These correspond to the coarse granular capillary wall deposits of IgG and C3 on IF in acute post-strep GN [63]. EM in post-infectious GN also usually shows mesangial and subendothelial deposits, but the hallmark “humps” clinch the diagnosis [64]. Other infection-related GN (staphylococcal, etc.) can have similar deposits [65].

MPGN describes a pattern of capillary wall double-contouring on LM with mixed Ig and complement deposition [9]. EM in immune-complex MPGN (formerly MPGN type I or III) typically shows subendothelial deposits and mesangial interposition - mesangial cell processes intermixing within a splitting GBM [66]. EM is important to distinguish immune-complex MPGN from the complement-mediated C3 glomerulopathies (discussed next), which can have a similar LM appearance but different ultrastructural deposits [67].

C3 glomerulopathy and dense deposit disease

C3G is a recently defined category of complement-mediated glomerular disease in which C3 (and complement factors) dominate the deposits, with minimal or no immunoglobulin [68]. Two major subtypes are recognized: dense deposit disease (DDD) and C3 glomerulonephritis (C3GN) [69]. These entities often show an MPGN pattern by LM and strong C3 staining by IF, but EM is essential to differentiate DDD from C3GNnephjc.com [70].

In dense deposit disease, EM reveals pathognomonic dense osmiophilic material within the GBM [71]. The deposits are often ribbon-like or sausage-shaped, appearing as a continuous, extremely electron-dense transformation of large segments of the GBM [72]. Instead of discrete granular deposits, the lamina densa is replaced by a homogeneous dense material, which can extend for long stretches [73]. These intramembranous dense deposits give DDD its name and are typically intramembranous (within GBM), but can also involve mesangial areas and Bruch’s membrane in the eye (hence the older name “dense deposit disease” or MPGN type II) [74]. C3GN, on the other hand, shows C3-dominant deposits that are more heterogeneous in shape and density [75]. EM in C3GN may show scattered subendothelial or mesangial granular deposits that are not uniformly electron-dense [76]. The GBM in C3GN does not undergo the confluent dense transformation seen in DDD [77]. There may be subepithelial hump-like deposits in some C3GN cases, similar to infection-related GN, but the key is that the deposits, though electron-dense, still look like “immune complexes” (amorphous, not replacing the GBM matrix) [78]. In contrast, DDD’s deposits are so dense and band-like that the normal trilaminar GBM structure is obliterated [79].

Practically, a renal pathologist relies on EM to confirm a diagnosis of DDD: IF might alert us by showing C3-only staining, but only EM can visualize the signature dense ribbon deposits [80]. This distinction matters because DDD and C3GN have different genetic associations and clinical courses [69]. DDD often involves genetic or autoantibody-related dysregulation of the alternative complement pathway (C3 nephritic factors, factor H mutations, etc.) and carries a high risk of recurrence in transplants [81]. C3GN is more heterogeneous [82]. Both are treated with complement-targeted therapies in some cases, underscoring the need for accurate classification [82]. EM also helps exclude masqueraders - for example, some cases of infection-associated MPGN can show bright C3 and even dense deposits, but careful EM exam might reveal mixed Ig or substructure pointing to cryoglobulin, etc. [83].

Hereditary nephritis: Alport syndrome and thin basement membrane nephropathy

Ultrastructural examination of the GBM is crucial for diagnosing hereditary collagen IV nephropathies [84]. Alport syndrome is caused by mutations in type IV collagen (most often X-linked COL4A5), leading to progressive nephritis with hearing loss [85]. Thin basement membrane nephropathy (TBMN) (also called thin GBM disease) is usually due to heterozygous COL4A3/A4 mutations and causes isolated hematuria with diffusely thin GBMs [85]. While genetic testing is now available, many cases are first detected or confirmed by classic EM findings in the kidney biopsy [1].

In Alport syndrome, the cardinal EM feature is an irregular, alternating thinning and thickening of the GBM with areas of longitudinal splitting and lamellation of the lamina densa [86]. Early in Alport (young children), the GBM may be uniformly thinned (hence easily missed or confused with TBMN) [87]. But with age, the GBMs in Alport patients develop foci of marked thickening with a laminated “basket weave” appearance - multiple layers of disrupted basement membrane often with a scalloped, festooned contour [88]. Under EM, one sees the GBM broken into an array of interweaving strands of collagenous material, sometimes with small electron-lucent areas or microspheres within (representing defective collagen meshwork) [89]. A classic description is “basket-weave” GBM due to diffuse lamellation [84]. Measurements show areas of GBM can be extremely thick (600-800 nm) adjacent to areas extremely thin (<150 nm) [90]. Podocyte foot process effacement is often present, especially in later stages when proteinuria is heavy [91]. Another finding in Alport GBM can be “granular” or microgranular inclusions and small “breadcrumb” densities between lamellae - these are not immune deposits but rather components of the abnormal matrix [92]. EM changes are usually global (affecting all glomeruli) in X-linked Alport by adolescence, whereas carriers may show segmental areas of lamellation [93]. Importantly, EM is diagnostic in classic Alport, as LM findings (foam cells, FSGS scars) are not specific, and IF is typically negative [93]. When the EM changes of a collagenopathy are seen, genetic testing for COL4A3-5 should be pursued [94].

TBMN is essentially the extreme mild end of the collagen IV disorder spectrum [95]. EM here shows diffuse, uniform thinning of the GBM without any lamellation or architectural distortion [96]. An adult normal GBM is ~300-400 nm thick; in TBMN, GBM thickness is markedly reduced [96,97]. A generally accepted diagnostic threshold is GBM thickness <250 nm in adults (and <180 nm in children) in at least 50% of sampled capillaries [98]. By EM, the GBM in TBMN retains a smooth single-layered appearance (trilaminar structure with intact lamina densa) but is attenuated throughout, often averaging ~150-250 nm in thickness [99]. There should be no significant lamellation, splitting, or “basket weave” change, which distinguishes TBMN from early Alport [100]. Essentially, TBMN looks like a normal GBM, just thinner [100,101]. No electron-dense deposits are present (unless there is a superimposed IgA or other disease) [102]. In patients with isolated hematuria and normal LM, finding uniformly thinned GBMs on EM secures the diagnosis of TBMN [98]. This prevents mislabeling the patient as “IgA nephropathy” or other cause of hematuria in cases where IF may show incidental IgM or IgA staining but the true lesion is thin GBM [98].

It is worth noting that TBMN often results from heterozygous mutations in the same collagen genes that cause Alport when biallelic [103]. There is an overlap: some “TBMN” patients (especially those with COL4A3/A4 mutations) can slowly progress to chronic kidney disease or have family members with Alport [104]. EM cannot always separate an isolated TBMN from a mild Alport phenotype, but the absence of any basket-weave changes or abnormal focal thickening strongly favors TBMN [105]. Genetic correlation is helpful in those cases [105].

Amyloidosis and fibrillary/immunotactoid glomerulopathies

When pathologic deposits are present in glomeruli, EM can reveal whether they are organized fibrils or microtubules, which is decisive for diagnosis [106]. Renal amyloidosis (usually AL or AA type) is characterized by the deposition of amyloid fibrils in the mesangium, capillary walls, and vessel walls [107]. By LM, amyloid is identified by Congo red stain and apple-green birefringence; IF may or may not identify a light chain [107]. EM in amyloidosis is pathognomonic: it shows randomly oriented, non-branching fibrils 8-12 nm in diameter coursing through the mesangial matrix or expanding the GBM [108]. These fibrils are rigid, of uniform thickness (~10 nm), and form felt-like masses without any microtubular structure [108]. EM confirmation of these fibrils is the gold standard for diagnosing amyloid [109]. In difficult cases (e.g., a doubtful Congo red stain or uncommon amyloid protein types), seeing these fibrils clinches the diagnosis of amyloidosis [110]. EM can also localize amyloid to specific compartments (e.g., identifying predominantly vascular vs glomerular deposition) [111]. All amyloid types (AL, AA, etc.) share the fibrillar EM appearance, so additional typing (by immuno-EM or mass spectrometry) is required to determine the protein, but the presence of fibrils itself differentiates amyloid from other fibrillar deposits like fibrillary GN [112].

Fibrillary glomerulonephritis (FGN) and immunotactoid glomerulopathy (ITG) are rare idiopathic glomerular diseases characterized by non-amyloid, organized deposits [113]. Distinguishing them from amyloid and from each other relies heavily on EM [113]. In fibrillary GN, EM shows randomly arranged fibrils with an average diameter of about 18-20 nm (range roughly 10-30 nm) [113]. These fibrils resemble amyloid in being non-branching filaments, but they are approximately twice the thickness of amyloid fibrils [114]. They do not form the beta-pleated sheet structure that gives Congo positivity, hence the Congo red stain is negative in FGN [115]. The fibrils in FGN are typically localized to the mesangium and along capillary walls, often causing mesangial expansion and a membranoproliferative pattern [116]. IF in FGN usually shows polyclonal IgG (often IgG4) and C3 in the deposits. A key recent discovery is that a protein called DNAJB9 is specifically associated with FGN deposits, and its detection (by IHC or mass spec) can serve as a diagnostic marker, sometimes even obviating the need for EM if positive [117]. But EM remains the definitive test for FGN in practice - seeing those 20 nm fibrils confirms the diagnosis [117].

ITG is much rarer and is characterized by larger microtubular structures in the deposits [118]. EM in ITG shows cylindrical microtubules with hollow centers, often arranged in parallel arrays or stacks (hence “tactoids,” reminiscent of microtubule bundles) [119]. The diameter of immunotactoid microtubules is typically 30-50 nm (range can be 20-90 nm), markedly larger than fibrillary GN fibrils [120]. These structures may appear as circles or polygons in cross-section (hollow cores visible) and as rods in longitudinal section [121]. IF in ITG usually reveals a monoclonal immunoglobulin (often IgG κ or λ), and there is an association with lymphoproliferative disorders [122]. By LM, ITG can look like FGN or MPGN, so EM is required to demonstrate the microtubules [122].

Electron microscopy in renal transplant pathology

In kidney transplant biopsies, EM has emerging importance for both recurrent diseases and allograft-specific pathologies [123]. Many recurrent glomerulonephritides in the graft manifest ultrastructural changes identical to the native disease (e.g., recurrent IgA nephropathy will show mesangial deposits on EM, recurrent FSGS will show podocyte effacement, etc.) [123]. Thus, EM can help confirm recurrent disease, sometimes before it is fully developed by LM [124]. A prime example is recurrent FSGS in a transplant: an early recurrence may have normal LM and negative IF, but EM can detect diffuse foot process effacement, alerting clinicians to aggressive FSGS relapse even before sclerosis forms [125]. Similarly, recurrent membranous in a transplant can be recognized by EM detection of subepithelial deposits, even if IF for PLA2R or IgG is not done on the biopsy [126].

Beyond recurrent diseases, transplant glomerulopathy (TG) is a chronic allograft injury pattern usually due to chronic antibody-mediated rejection [127]. TG by LM shows a double-contour (duplicated GBM) appearance in glomerular capillaries, often with mesangial interposition (an MPGN-like change) [128]. EM is very sensitive in detecting early transplant glomerulopathy - ultrastructurally, TG is characterized by multilamination of the GBM (new layers of basement membrane material laid down, with an expanded electron-lucent zone between layers) [129]. In early stages, EM might show two to three layers to the GBM (“onion-skinning”) even when LM is not yet showing obvious double contours [130]. Endothelial cells may be swollen, and the subendothelial space widened by fluffy material on EM [131]. This allows detection of chronic antibody-mediated injury months or years before overt changes accrue on LM [132]. Recognizing early TG can be clinically significant, as it might prompt intensification of immunosuppression or therapy for donor-specific antibodies to stave off progression [132].

Another ultrastructural marker in chronic rejection is peritubular capillary basement membrane multilayering - EM of peritubular capillaries shows five or more concentric layers of basement membrane, which is a diagnostic feature of chronic antibody-mediated rejection even without apparent light microscopic changes [133]. Some centers now perform EM on protocol transplant biopsies to look for such changes, as recommended by Banff criteria (the international consensus on transplant pathology) [123].

Banff guidelines indeed recommend increased use of EM in renal allograft biopsies, not only for suspected recurrent or de novo glomerular diseases, but specifically to identify early TG lesions before LM changes are overt [134]. A Banff report in 2013 highlighted that EM can unmask incipient TG when C4d staining or donor-specific antibody data alone are inconclusive [134].

Moreover, some rare entities in transplants (e.g., light chain deposition disease, fibrillary GN, etc., occurring de novo) may only be definitively diagnosed with EM and IF [135]. Viral infections like CMV or BK virus can sometimes be identified by their ultrastructural inclusions on EM of a transplant biopsy, though in practice, IHC and PCR have largely supplanted EM for viral detection [136].

Diagnostic yield and clinical impact of EM

A central question in renal pathology is how often EM alters the diagnosis or influences clinical management in kidney biopsies [137]. Decades of studies consistently demonstrate that EM provides a substantial diagnostic yield [138]. Early reports from the 1990s, such as those by Pearson et al. (1994) and Haas (1997), found that EM contributed important diagnostic information in approximately 75% of cases and was essential in about 20-25% [139,140]. Subsequent studies in the 2000s and 2010s have yielded comparable results. For instance, Mokhtar et al. (2011) reported that EM provided significant input in 39% of 273 biopsies and was essential in 17%, particularly in diagnosing MCD, hereditary nephritis (Alport syndrome), FGN, and certain LN classes [141]. Similarly, Elhefnawy et al. (2015) found that EM provided useful information in around 66% of 120 biopsies and was essential in 25% [142]. More recently, a 2019 series from the American University of Beirut Medical Center (AUBMC) demonstrated that EM altered the primary diagnosis in 23% of 150 cases and contributed a secondary diagnosis in another 23%, being particularly valuable in identifying thin GBM lesions, MCD, and MN [143].

The diagnostic yield of EM is especially high in scenarios where LM and IF are normal or nonspecific, such as in cases of unexplained nephrotic syndrome (to distinguish MCD from FSGS), isolated hematuria (to differentiate IgA nephropathy from thin GBM disease), or suspected organized deposit diseases (to distinguish amyloidosis from fibrillary GN) [144]. EM is also indispensable when differentiating between morphologically similar entities, including subtypes of C3G or lupus MN versus primary MN [145]. In these situations, EM often provides the decisive ultrastructural evidence that clinches the diagnosis. Table 2 outlines the typical EM signatures of major immune complex-mediated glomerulonephritides.

**Table 2:** Ultrastructural features of immune complex-mediated glomerulonephritis (GN)

Disease	Deposit Location	Glomerular Basement Membrane (GBM) Response	Characteristic EM Finding
MPGN [66]	Subendothelial, mesangial	Double contours	Mesangial interposition
IgA nephropathy [146]	Mesangial ± subendothelial	Mild expansion	Patchy dense deposits
Lupus nephritis [147]	Mesangial, subendothelial ± subepithelial	GBM duplication	Tubuloreticular inclusions
Postinfectious GN [148]	Subepithelial	Endocapillary proliferation	Hump-like deposits

Across published series, the proportion of renal biopsies in which EM provides decisive diagnostic information ranges between 17% and 25%, while supplemental contributions occur in up to 75% of cases [139-143]. In contrast, common entities such as IgA nephropathy are primarily diagnosed by IF, where EM has limited incremental value. Moreover, Table 3 provides a summary of characteristic EM findings across major glomerular diseases.

**Table 3:** Summary of characteristic electron microscopy (EM) findings across major glomerular diseases C3GN, C3 glomerulonephritis; DDD, dense deposit disease; FSGS, focal segmental glomerulosclerosis; GBM, glomerular basement membrane

Disease Category	Key EM Finding(s)	Diagnostic Significance
Minimal change disease	Diffuse podocyte foot process effacement	Confirms podocytopathy; distinguishes from early FSGS
Focal segmental glomerulosclerosis	Segmental effacement, GBM wrinkling	Identifies primary vs secondary FSGS
Membranous nephropathy	Subepithelial electron-dense deposits, GBM spikes	Defines stages I-IV; confirms diagnosis
IgA nephropathy	Mesangial ± subendothelial deposits	Localizes immune complexes
Lupus nephritis	Full-house deposits, tubuloreticular inclusions	Guides class differentiation
C3 glomerulopathy	Dense intramembranous or subendothelial C3 deposits	Differentiates DDD vs C3GN
Alport syndrome	GBM thickening, thinning, and lamellation	Diagnostic of collagen IV nephropathy
Thin basement membrane nephropathy	Uniform GBM thinning without lamellation	Confirms benign hematuria
Amyloidosis	Random, non-branching fibrils (8-12 nm)	Confirms amyloid deposition
Fibrillary glomerulonephritis (GN)	Random fibrils (18-20 nm)	Distinguishes from amyloid (Congo-negative)
Immunotactoid GN	Microtubules (30-50 nm), often parallel	Suggests monoclonal process
Transplant glomerulopathy	GBM multilamination, endothelial swelling	Indicates chronic antibody-mediated injury

Current debates and challenges

Despite its unparalleled diagnostic value, the use of EM in renal pathology continues to face significant practical and institutional challenges [1]. The foremost concern is cost and declining availability [1]. Establishing and maintaining an EM facility requires expensive instrumentation, electron microscopes, ultramicrotomes, and vacuum systems, as well as reagents such as glutaraldehyde and osmium tetroxide [149]. Moreover, trained EM technologists are increasingly scarce, as few centers continue to provide formal training in tissue processing and ultrathin sectioning [150]. In many hospitals, particularly in resource-limited regions, EM is no longer routinely available, and kidney biopsy specimens must be referred to external reference laboratories [151]. While this selective use reduces immediate expenses, it can compromise diagnostic accuracy and delay treatment decisions [151]. Many experts argue that the potential cost of misdiagnosis far outweighs the operational savings gained by omitting EM [151]. Moreover, accurate EM interpretation requires substantial experience and familiarity with normal ultrastructure and potential artifacts [152]. However, the number of proficient renal pathologists in EM has declined as training programs increasingly prioritize molecular techniques [153]. Misinterpretation, such as mistaking sectioning artifacts for immune deposits, can lead to diagnostic errors [154]. Some institutions have responded by designating dedicated ultrastructural pathologists or seeking external consultation from reference centers with specialized expertise [154]. Time constraints also limit EM utilization [155]. Traditional processing and embedding protocols can take several days, which delays reporting, an issue particularly relevant in acute glomerulonephritis or transplant rejection, where clinicians expect rapid turnaround [156]. Although accelerated “fast EM” protocols can produce results within 24-48 hours, such workflows are not universally available [157]. Another evolving issue is the perceived redundancy of EM in the era of molecular diagnostics [158]. The development of serologic and genetic markers, such as anti-PLA2R antibodies for MN and COL4A gene testing for Alport syndrome, has led some clinicians to question EM’s necessity [158]. Nevertheless, EM remains crucial in seronegative or atypical cases, and in identifying secondary forms of MN or mixed patterns that molecular tests may miss [159]. Moreover, in many parts of the world, genetic testing is less accessible or more expensive than EM, reinforcing EM’s continued relevance as a practical diagnostic tool [160].

Despite the undeniable diagnostic advantages of EM, its routine use must be balanced against cost, time, and resource limitations [159]. Several cost-effectiveness analyses indicate that targeted use of EM, rather than blanket application to all renal biopsies, provides the best balance between diagnostic yield and expenditure [159]. Selective deployment (guided by clinical context, LM/DIF results, and disease suspicion) minimizes unnecessary costs while preserving diagnostic accuracy, especially in resource-limited centers [160].

Moreover, despite its diagnostic value, EM is not universally applicable or sufficient in all renal biopsy contexts [1]. For example, amyloid subtyping cannot be achieved by EM alone, as ultrastructural appearance is similar across amyloid types; mass spectrometry-based proteomic analysis remains essential for definitive typing [89]. Likewise, EM cannot replace IHC or molecular testing in disorders such as monoclonal immunoglobulin deposition disease or hereditary nephropathies [90]. In the field of renal transplantation, EM is not formally required by current KDIGO or Banff guidelines for allograft evaluation, although it can detect early ultrastructural changes in transplant glomerulopathy and antibody-mediated rejection before light microscopic alterations appear [124]. Recognizing these limitations underscores the complementary rather than exclusive role of EM in renal pathology.

Integration with newer tools: AI, molecular pathology, and immuno-EM

Renal pathology is rapidly evolving, and EM is now being enhanced rather than replaced by new technologies [161]. AI has emerged as a major innovation, streamlining EM image analysis [162]. Manual assessment of GBM thickness and podocyte foot process effacement is laborious and subjective, but recent AI models, such as the multi-center “TEM-AID” system, can automatically segment glomerular structures and classify disease subtypes from EM images with accuracy comparable to expert pathologists [162]. Molecular and proteomic techniques also complement EM in diagnostic precision. Biomarkers such as PLA2R in MN and DNAJB9 in FGN now provide molecular confirmation that reinforces EM findings [163]. Similarly, mass spectrometry-based amyloid typing allows precise identification of amyloid subtypes, while EM confirms the presence of fibrillar deposits [164]. Genetic testing for COL4A mutations has transformed the diagnosis of Alport syndrome and TBMN, often guided by EM findings that prompt targeted sequencing [165]. Finally, immunoelectron microscopy (IEM) offers ultrastructural localization of antigens using immunogold labeling [166]. Though technically demanding, it has clarified difficult cases such as masked immune complex disease, monoclonal deposition disorders, and antigen localization in MN [167]. Future developments, including quantum dot tagging and correlative light-electron microscopy, promise broader clinical application [168].

## Conclusions

EM remains an essential component of renal biopsy interpretation, providing ultrastructural insight that complements LM and IF. Its ability to localize immune deposits, characterize GBM alterations, and define podocyte and endothelial changes makes it indispensable for the accurate classification of glomerular diseases. From confirming podocytopathies such as MCD and FSGS, to differentiating membranous and immune complex glomerulonephritides, and diagnosing hereditary collagen IV nephropathies, EM bridges morphology with molecular pathology. Although genetic and serologic techniques continue to advance, they cannot fully replace the diagnostic precision and morphological depth offered by EM. Quantitative synthesis of EM yield across biopsy cohorts may further clarify when its application is most cost-effective and clinically justified. Continued integration of EM findings with clinical and molecular data will remain vital for advancing diagnostic nephropathology and improving patient care.
